# The Impact of Inadequate Health Literacy in a Population with Musculoskeletal Pain

**DOI:** 10.3928/24748307-20181101-01

**Published:** 2018-12-06

**Authors:** Rosie J. Lacey, Paul Campbell, Martyn Lewis, Joanne Protheroe

## Abstract

Musculoskeletal conditions are a major cause of ill health and disability. Inadequate health literacy may partly explain why musculoskeletal self-management programs are not effective for some patients. This study prospectively evaluated the impact of patients' health literacy level on their musculoskeletal pain and physical function (PF) following usual primary care. Primary care patients (*N* = 4,720) who had consulted for musculoskeletal pain were mailed a baseline questionnaire; responders were sent a 6-month follow-up questionnaire. The measure of health literacy used was the single-item Literary Screener at baseline, and the outcomes were PF and pain intensity at the 6-months follow-up. Analysis was conducted by linear regression. The number of patients who responded was 1,890 (40%); 17.3% (95% CI [15.6%–19%]) of them had inadequate health literacy. Inadequate health literacy was associated with older age (*p* < .05), lower education, mental health, and comorbidities (all *p* < .001), but not by gender (*p* = .642). At the 6-month follow-up stage, patients with inadequate health literacy had lower PF (mean difference −12.2; 95% CI [−16.7, −7.6]) and higher pain intensity (mean difference 1; 95% CI [0.6, 1.4]), which was adjusted for age, gender, education, mental health, and comorbidities. Differences in PF and particularly pain scores between patients with inadequate and adequate health literacy increased over 6 months. Future studies should develop interventions that better support patients who have musculoskeletal pain with inadequate health literacy to successfully manage their pain. **[*HLRP: Health Literacy Research and Practice*. 2018;2(4):e214–e220.]**

Musculoskeletal conditions are a major cause of ill health and disability worldwide, with substantial impacts on patients' quality of life and health care resource use (Woolf & Pfleger, 2003). Musculoskeletal conditions, including osteoarthritis, are generally considered to be long-term conditions, for which the mainstay of treatment is supported self-management. However, a recent review of self-management education programs for osteoarthritis concluded that these programs conferred “little or no benefit” for self-management skills, or health outcomes (Kroon et al., 2014). Self-management programs require patients to have a high level of participation and engagement (Adams, 2010). There is growing evidence that factors related to health equity (e.g., socioeconomic disadvantage, inadequate health literacy) may be partly the reason that some patients benefit less from musculoskeletal self-management interventions (Beneciuk et al., 2017; Kapoor, Eyer, & Thorn, 2016).

Health literacy refers to the personal characteristics and social resources needed for people and communities to access, understand, appraise, and use information and services to make decisions about health (Dodson, Good, & Osborne, 2015). People with low socioeconomic status or low levels of education are more likely to have poorer health literacy (European Health Literacy Project Consortium, 2012), and this is associated with poorer health outcomes, poorer use of health care services (Berkman, Sheridan, Donahue, Halpern, & Crotty, 2011), and impacts on self-management skills (Mackey, Doody, Werner, & Fullen, 2016). Evidence from subgroup analyses in a review of self-management education programs for osteoarthritis showed that some outcomes differed according to factors associated with health literacy (e.g., education level; Kroon et al., 2014). However, only 14% of included trials provided information on participants' health literacy, leading Kroon et al. (2014) to suggest that future intervention development for self-management programs should consider patient health literacy to explore issues of health equity.

Few studies have investigated the effect of health literacy specifically on musculoskeletal pain and physical function (PF). A cross-sectional study of adults age 60 years and older found that those with low health literacy had a significantly higher prevalence of arthritis (Kim, 2009), and emerging evidence suggests that health care professionals find pain management in patients with low health literacy challenging, as these patients have less understanding and less control of their pain (Adams et al., 2016). However, no research to date has considered the prospective effect of health literacy on outcomes for those with musculoskeletal pain; this is needed to inform interventions that better meet the needs of patients with low health literacy. The aim of this study is to prospectively evaluate the impact of patients' health literacy level on their musculoskeletal pain and PF outcomes after a primary care consultation.

## Methods

We conducted secondary data analysis of the Keele Aches and Pains Study (KAPS), a prospective cohort study in 14 United Kingdom (UK) primary care practices. Full details of the protocol have been published (Campbell et al., 2016). Ethical approval for the KAPS was granted by the South East Scotland Research Ethics Committee, UK (14/SS/0083).

Consecutive patients age 18 years and older who visited their family doctor with 1 or more of 5 musculoskeletal pains (back, neck, shoulder, knee, or multisite pain), including chronic and acute pain, were invited to take part in the study. Inclusion criteria were patients registered at participating general practices, age 18 years or older, consulting with the included musculoskeletal pain presentations, and able to read and understand English. Exclusion criteria were indication of a serious pathology (e.g., suspected fracture, cancer), inflammatory arthritis, crystal disease, spondyloarthropathy, polymyalgia rheumatica, pregnancy-related pain problems, urgent cases (e.g., cauda equina syndrome), or vulnerable patients (e.g., experienced recent trauma, cognitive impairment, dementia, or terminal illness). There was no intervention in this cohort study, and patients received usual care from their family doctor. Eligible patients (*N* = 4,720) were mailed a study pack (including an information sheet and baseline questionnaire) from their family doctor shortly after their musculoskeletal pain consultation. Information regarding the study included that completion and return of the baseline questionnaire would signify participants' willingness to take part and receive a follow-up questionnaire. All patients who consented to participate were mailed 6-month follow-up questionnaires. Nonresponders at both stages were mailed reminders at 2 weeks and repeat questionnaires 2 weeks later.

### Outcome Measures

PF and pain intensity were both measured in baseline and in the 6-month questionnaires. PF was measured using the Physical Functioning subscale of Short Form-36 (SF-36 PF), which consists of 10 items; scores range from 0 to 100, with lower scores indicating worse health (Ware, 2000). Three pain intensity questions specifically asked about the aches, pain, or stiffness that patients had visited their doctor about (current pain; average usual pain in last 2 weeks; and least pain in last 2 weeks), each on a 0 to 10 numerical rating scale, 0 indicating no pain, 10 indicating pain as bad as it could be (Campbell et al., 2016; Deyo et al., 2015).

### Predictor Variable

Health literacy was measured at baseline using the single-item literacy screener (SILS): “How often do you need to have someone help you when you read instructions on pamphlets, or other written material from your doctor or pharmacy?” (Morris, MacLean, Chew, & Littenberg, 2006). Response options: *often*, *always*, *sometimes*, *rarely*, *never*.

***Potential confounding variables (measured at baseline)***. Three stages of education: “How old were you when you left school?” (years); “Did you go into full-time education (college or university)?” (yes, no); “Have you gained qualifications through study as an adult?” (yes, no) (Campbell et al., 2016).

***Comorbidities***. Diabetes; breathing problems/chronic pulmonary obstructive disease/asthma; heart problems/high blood pressure; chronic fatigue syndrome/myalgic encephalomyelitis/fibromyalgia; anxiety/depression/stress; and other (Campbell et al., 2016).

***Mental health***. Mental component summary score of SF-36 (Ware, 2000).

### Statistical Analysis

Characteristics of the study population were analyzed according to level of health literacy, using the one-way analysis of variance trend test with linear contrast (1 *df*). Associations between health literacy, and PF or pain intensity (average of the three pain intensity scores) were analyzed using linear regression (adjusted for age, gender, three stages of education, comorbidities, mental health). For regression analyses, health literacy was dichotomized into inadequate health literacy (*often*, always, *sometimes need help*) and adequate health literacy (*rarely, never need help*) as used previously (Morris et al., 2006). Results are presented as mean differences (MD) with 95% confidence intervals [95% CI], and standardized MD, i.e., effect size relative to baseline standard deviation of 28.7 (SF-36 PF) and 2.37 (pain) (Cohen, 1988). Effect sizes were interpreted as suggested by Cohen (1988): 0.2 “small,” 0.5 “moderate,” 0.8 “large.” To give context, the percentage change in PF and pain scores were calculated (MD at baseline or 6 months per mean score for study population).

## Results

In total, 40% of patients (*n* = 1,890) consented to the baseline invitation. The mean age of participants was 58.3 years (range, 18 to 98 years), and 60.6% were female. At the 6 month follow-up, 1,452 patients responded (76.8%). No differences were found between responders and nonresponders at 6-months for baseline gender, later stages of education, PF, or comorbidities **(Table A)**. Nonresponders at 6 months were more likely to have left school earlier, have inadequate health literacy (25% vs. 15%), higher pain score, poorer mental health, and be younger than responders.

In **Table 1**, 17.3% (95% CI [15.6%–19%]) of patients reported inadequate health literacy. Inadequate health literacy was associated with older age (60.2 years vs. 57.9, *p* < .05), lower education (all stages), poorer mental health, and comorbidities (all *p* < .001), but not gender (*p* = .642).

At baseline, patients with inadequate health literacy had lower PF and higher pain scores than those with adequate health literacy, and these associations remained after adjustment for age, gender, and all education stages (**Table 2**). The difference in PF and pain scores between health literacy groups was reduced after additional adjustment for comorbidities and mental health but remained significant (*p* < .001).

At the 6-month follow-up, patients with inadequate health literacy at baseline had significantly lower PF (MD = −22.2, 95% CI [−27.1, −17.4]; *p* < .001) and higher pain (MD = 1.79, 95% CI [1.35, 2.24]; *p* < .001) scores after adjustment for age, gender, and all education stages than those with adequate health literacy with large effect sizes (PF = −0.77, 95% CI [−0.94, −0.61]; *p* < .001; pain: 0.76, 0.57, 0.95; *p* < .001; Cohen, 1988; **Table 2**). Additional adjustment for comorbidities and mental health reduced the difference in PF (MD: −12.2, −16.7, −7.6) and pain (MD: 0.99, 0.56, 1.41) scores between the health literacy groups, and effect sizes for PF (−0.42, −0.58, −0.26) and pain (0.42, 0.24, 0.59) to small to moderate. The difference between the health literacy groups remained larger at 6 months than at baseline, particularly for pain (24% higher pain at 6 months vs. 12% higher at baseline) for inadequate compared to adequate health literacy.

## Discussion

To our knowledge, this is the first prospective observational study to provide evidence that health literacy level has an impact over time on musculoskeletal pain and PF in primary care patients. Six months after consulting their family doctor for musculoskeletal pain, differences in PF and particularly pain scores between patients with inadequate and adequate health literacy had increased, suggesting that those with poor health literacy benefit less from current primary care management strategies. Adjustment for potential confounders reduced the effect sizes between those with inadequate and adequate health literacy, although the differences remained significant representing 23% lower PF and 24% higher pain at 6 months for those with inadequate compared to adequate health literacy.

Our results contrast with the findings from a systematic review, which found no consistent association between low health literacy and poorer functional outcomes in patients with chronic musculoskeletal conditions (Loke et al., 2012). One included study reported an association between low health literacy, and more pain and functional limitation (Kim, 2009), although Loke et al. (2012) identified many methodological weaknesses in the included studies. A UK back pain trial reported that participants with low socioeconomic status (based on occupation) benefitted less from a prognostic stratified care intervention for low back pain than those with high socioeconomic status (Beneciuk et al., 2017). Our results may partly explain these findings. Indeed, Beneciuk et al. (2017) suggested that barriers to good health outcomes experienced by patients with low socioeconomic status, such as low health literacy, may have influenced their results.

Little evidence exists for the impact of low health literacy on self-management skills for musculoskeletal conditions, although a recent preliminary study of patients with chronic pain at clinics that serve patients with low socioeconomic status found that lower levels of health literacy were associated with greater catastrophizing and lower pain-related self-efficacy (Kapoor, Eyer, & Thorn, 2016). A systematic review of the effectiveness of educational interventions in people with low literacy levels showed a modest effect on knowledge and self-efficacy, although there was a lack of high-quality evidence (Lowe et al., 2013). We support the authors' recommendation that future patient education interventions for musculoskeletal conditions should recruit and engage people with lower levels of literacy.

This study has several strengths. We used a large, prospective cohort of musculoskeletal consulters in primary care. We used a validated health literacy screening measure (SILS) because it is a short, simple measure developed from the 16-item Short Test of Functional Health Literacy in Adults (Baker, Williams, Parker, Gazmararian, & Nurss, 1999), suitable for postal questionnaires (Morris et al., 2006). We adjusted for several potential confounders (sociodemographic factors, educational history, comorbidities, and mental health). There are some limitations to this study. The SILS is a screening test and not a direct measure of health literacy, although it was developed to efficiently identify patients who need help reading health-related materials (Morris et al., 2006). In our study, the ability to read and understand English could have excluded patients on the basis of their functional health literacy. Nonresponders at 6-month follow-up were more likely to have baseline inadequate health literacy than responders, which may have resulted in an unavoidable underestimate of low health literacy in this cohort. This is supported by our prevalence of low health literacy (17%) being less than a general population interview survey suggests (43%–61%; Rowlands et al., 2015). Response to our study was 40%, although retention in the cohort was good at 6 months. Forty percent is a moderate response, although similar mean pain intensity values and other baseline characteristics are reported in other primary care consultation musculoskeletal cohort studies (Dunn, Jordan, & Croft, 2006) with higher response rates. Misclassification of outcomes could have occurred if responders to the questionnaires did not answer the PF and pain questions in relation to their aches, pain, or stiffness, but to pain more generally. However, extensive work with our patient and public Research User Group resulted in the term “aches, pain, or stiffness” being used for musculoskeletal pain in our questionnaires. Further limitations may be the lack of information on race or ethnicity, socioeconomic status, income and body mass index, as low health literacy is reported to be associated with these factors (European Health Literacy Project Consortium, 2012; Geboers, Reijneveld, Jansen, de Winter, 2016; Sperber et al., 2013). There is scope to investigate these and other potential confounders (e.g., treatments and medications, duration of pain) in future research.

This study has shown that primary care patients' health literacy level affects their musculoskeletal outcomes after seeing their family doctor; differences in PF, and particularly pain, between patients with inadequate and adequate health literacy increase over 6 months. We suggest that the disappointing results of self-management approaches for patients with musculoskeletal pain may be partly explained by low health literacy. Future studies should develop interventions that better support patients with musculoskeletal pain with low health literacy to successfully manage their pain.

## Figures and Tables

**Table 1:** Study Population by Baseline Health Literacy Response Categories

**Characteristic**	**Never**	**Rarely**	**Sometimes**	**Often**	**Always**	**Adequate**^a^	**Inadequate**^a^	**Total**

Overall, *n* (%)	1,321 (70.3)	234 (12.4)	185 (9.8)	72 (3.8)	68 (3.6)	1,555 (82.7)	325 (17.3)	1,880^b^

Age (years), *M* (*SD*)^*^	57.4 (15.6)	60.5 (16.7)	61.2 (17.5)	59.1 (16.2)	58.5 (17.7)	57.9 (15.8)	60.2 (17.2)	58.3 (16.1)

Gender, *n* (%)								
	
Female	812 (61.5)	133 (56.8)	106 (57.3)	50 (69.4)	37 (54.4)	945 (60.8)	193 (59.4)	1,138 (60.5)
	
Male	509 (38.5)	101 (43.2)	79 (42.7)	22 (30.6)	31 (45.6)	610 (39.2)	132 (40.6)	742 (39.5)

Education, *n* (%)^**^ (age left school)								
	
≤ 16 years	1,009 (77.4)	198 (86.1)	159 (89.3)	54 (78.3)	62 (96.9)	1,207 (78.7)	275 (88.4)	1,482 (80.4)
	
≥ 17 years	294 (22.6)	32 (13.9)	19 (10.7)	15 (21.7)	2 (3.1)	326 (21.3)	36 (11.6)	362 (19.6)

Full-time education, *n* (%)								
	
No	857 (65.4)	188 (81)	152 (83.1)	56 (78.9)	60 (89.6)	1,045 (67.7)	268 (83.5)	1,313 (70.4)
	
Yes	454 (34.6)	44 (19)	31 (16.9)	15 (21.1)	7 (10.4)	498 (32.3)	53 (16.5)	551 (29.6)

Gained qualifications as an adult, *n* (%)^**^								
	
Yes	782 (61.3)	95 (43)	63 (36.2)	22 (32.4)	21 (32.3)	877 (58.6)	106 (34.5)	983 (54.5)
	
No	494 (38.7)	126 (57)	111 (63.8)	46 (67.6)	44 (67.7)	620 (41.4)	201 (65.5)	821 (45.5)

Comorbidities, *n* (%)^**^								
	
Yes	871 (66)	178 (76.1)	151 (81.6)	58 (80.6)	57 (83.8)	1,049 (67.5)	266 (81.8)	1,315 (70)
	
No	449 (34)	56 (23.9)	34 (18.4)	14 (19.4)	11 (16.2)	505 (32.5)	59 (18.2)	564 (30)

Mental health, *M* (*SD*)								
	
Baseline^**^	67.8 (21)	59 (20.8)	52.3 (21.7)	47.6 (23.9)	40.1 (25)	66.5 (21.2)	48.7 (23.3)	63.4 (22.6)
	
6 months^**^	73.4 (19.2)	67.2 (21.5)	59.1 (22.7)	55 (25.7)	46.7 (28.4)	72.5 (19.6)	56.8 (24.2)	70.3 (21)

Pain (average), *M* (*SD*)								
	
Baseline^**^	5 (2.3)	5.6 (2.2)	6.5 (2.1)	6.2 (2.3)	7 (2.2)	5.1 (2.3)	6.6 (2.2)	5.3 (2.4)
	
6 months^**^	3.9 (3)	4.7 (2.9)	5.7 (2.9)	5.9 (2.7)	6.7 (2.5)	3.9 (2.7)	5.8 (2.6)	4.1 (2.8)

Physical functioning, *M* (*SD*)								
	
Baseline^**^	53.7 (27.6)	45.3 (27.4)	32.7 (27)	32 (24.9)	33.3 (32.6)	52.4 (27.7)	32.7 (27.7)	49 (28.7)
	
6 months^**^	58.1 (29.5)	50.2 (29.7)	32.6 (27.9)	33.4 (26.9)	30.6 (33.2)	57 (29.6)	32.5 (28.2)	53.6 (30.6)

Note. *M* = mean difference.

a Inadequate health literacy = *often*, always, *sometimes need help;* adequate health literacy = *rarely, never need help*.

b *n* = 10; missing data for the health literacy question.

* *p* < .05.

** *p* < .001.

**Table 2:** Differences in Physical Function and Pain Intensity Scores Between Patients with Inadequate and Adequate Health Literacy^a^

**Characteristic**	**Unadjusted**			**Adjusted**^b^			**Adjusted**^c^		
		
	***M* [95% CI]**	**ES [95% CI]**	***M* [95% CI]**	**ES [95% CI]**	**% Change in Score**	***M* [95% CI]**	**ES [95% CI]**	**% Change in Score**

Physical function								
	
Baseline	−19.8 [−23.1, −16.4]	−0.69 [−0.80, −0.57]	−19.2 [−22.6, −15.7]	−0.67 [−0.79, −0.55]	−39.2	−9.5 [−12.8, −6.2]	−0.33 [−0.45, −0.22]	−19.4
	
6 months	−24.5 [−29.2, −19.7]	−0.85 [−1.02, −0.69]	−22.2 [−27.1, −17.4]	−0.77 [−0.94, −0.61]	−41.4	−12.2 [−16.7, −7.6]	−0.42 [−0.58, −0.26]	−22.8

Pain intensity (average)								
	
Baseline	1.49 [1.21, 1.77]	0.63 [0.51, 0.75]	1.28 [0.99, 1.57]	0.54 [0.42, 0.66]	24.2	0.65 [0.37, 0.94]	0.27 [0.16, 0.40]	12.3
	
6 months	1.96 [1.53, 2.40]	0.83 [0.65, 1.01]	1.79 [1.35, 2.24]	0.76 [0.57, 0.95]	43.7	0.99 [0.56, 1.41]	0.42 [0.24, 0.59]	24.1

Note. All tests of association were significant at *p* < .001. Mean difference from linear regression analyses calculated as mean score (inadequate HL group) minus mean score (adequate HL [reference group]). Percentage change in score calculated as mean difference at baseline or 6 months per mean score for study population. CI = confidence interval; ES = effect size; HL = health literacy; *M* = mean difference.

a Inadequate health literacy = *often*, *always*, *sometimes need help*; adequate health literacy = *rarely*, *never need help*.

b Adjusted for age, gender, age left school, further education, qualifications as adult.

c Additionally adjusted for baseline comorbidities and mental health score.

**Table A:** Responders and Nonresponders at the 6-month Follow-Up Questionnaire

**Characteristic**	**6-Month Follow-Up**	

	**Responders**	**Nonresponders**

Overall, *n* (%)	1,452 (76.8)	438 (23.2)

Inadequate HL, *n* (%)^a^		
	
Never/rarely	1,231 (85.1)	324 (74.8)
	
Sometimes/often/always	216 (14.9)	109 (25.2)
	
Age (years), *M* (*SD*)^a^	59.9 (15)	53.2 (18.3)

Gender, *n* (%)		
	
Female	867 (59.7)	278 (63.5)
	
Male	585 (40.3)	160 (36.5)

Education, *n* (%)^b^ (age left school)		
	
≤16 years	1,136 (79.3)	352 (84.2)
	
≥17 years	297 (20.7)	66 (15.8)

Full-time education, *n* (%)		
	
No	1,020 (70.9)	297 (69.1)
	
Yes	419 (29.1)	133 (30.9)

Gained qualifications as an adult, *n* (%)		
	
Yes	778 (55.7)	208 (50.5)
	
No	618 (44.3)	204 (49.5)

Comorbidities, *n* (%)		
	
Yes	1,027 (70.8)	289 (66.3)
	
No	423 (29.2)	147 (33.7)

Mental health, *M* (*SD*)^a^	64.9 (22.2)	58.5 (23.3)

Pain (average), *M* (*SD*)^a^	5.2 (2.4)	5.7 (2.4)

Physical functioning, *M* (*SD*)	49.4 (28.5)	47.8 (29.5)

Note. HL = health literacy.

a *p* < .001 by chi square test or independent samples t-test.

b *p* < .05.
